# Leucocyte Subsets Effectively Predict the Clinical Outcome of Patients With COVID-19 Pneumonia: A Retrospective Case-Control Study

**DOI:** 10.3389/fpubh.2020.00299

**Published:** 2020-06-18

**Authors:** Jiahua Gan, Jingjing Li, Shusheng Li, Chunguang Yang

**Affiliations:** ^1^Department of Urology, Tongji Hospital, Tongji Medical College, Huazhong University of Science and Technology, Wuhan, China; ^2^Department of Clinical Laboratory, Tongji Medical College, Union Hospital, Huazhong University of Science and Technology, Wuhan, China; ^3^Department of Intensive Care Unit, Tongji Medical College, Tongji Hospital, Huazhong University of Science and Technology, Wuhan, China

**Keywords:** SARS-CoV-2, COVID-19, prognosis, leucocyte, lymphocyte, cytokine

## Abstract

**Background:** The clinical characteristics of coronavirus disease 2019 (COVID-19) have been well-studied, while effective predictors for clinical outcome and research on underlying mechanisms are scarce.

**Methods:** Hospitalized COVID-19 pneumonia patients with definitive clinical outcome (cured or died) were retrospectively studied. The diagnostic performance of the leucocyte subsets and other parameters were compared using the area under the receiver operating characteristic curve (AUC). Further, the correlations between leucocyte subsets and inflammation-related factors associated with clinical outcome were subsequently investigated.

**Results:** Among 95 subjects included, 56 patients were cured, and 39 died. Older age, elevated aspartate aminotransferase, total bilirubin, serum lactate dehydrogenase, blood urea nitrogen, prothrombin time, D-dimer, Procalcitonin, and C-reactive protein levels, decreased albumin, elevated serum cytokines (IL2R, IL6, IL8, IL10, and TNF-α) levels, and a decreased lymphocyte count indicated poor outcome in patients with COVID-19 pneumonia. Lymphocyte subset (lymphocytes, T cells, helper T cells, suppressor T cells, natural killer cells, T cells+B cells+NK cells) counts were positively associated with clinical outcome (AUC: 0.777; AUC: 0.925; AUC: 0.900; AUC: 0.902; AUC: 0.877; AUC: 0.918, resp.). The neutrophil-to-lymphocyte ratio (NLR), neutrophil to T lymphocyte count ratio (NTR), neutrophil percentage to T lymphocyte ratio (NpTR) effectively predicted mortality (AUC: 0.900; AUC: 0.905; AUC: 0.932, resp.). Binary logistic regression showed that NpTR was an independent prognostic factor for mortality. Serum IL6 levels were positively correlated with leucocyte count, neutrophil count, and eosinophil count and negatively correlated with lymphocyte count.

**Conclusion:** These results indicate that leucocyte subsets predict the clinical outcome of patients with COVID-19 pneumonia with high efficiency. Non-self-limiting inflammatory response is involved in the development of fatal pneumonia.

## Introduction

In December 2019, a new type of β-coronavirus named the 2019 novel coronavirus (SARS-CoV-2) emerged in Wuhan, China, and spread rapidly throughout the world (1). As of May 31, 2020, 5.9 million cases of coronavirus disease 2019 (COVID-19) have been confirmed, including 367 thousand deaths (2). It was most likely initially a zoonotic infectious disease, but effective transmission between people was soon discovered (3). The clinical symptoms of SARS-CoV-2 infection are variable, including being asymptomatic, upper respiratory tract disease, viral pneumonia, fatigue, gastrointestinal symptoms, respiratory failure, and even death (4, 5). Its clinical characteristics have been well-evaluated, but effective predictors for clinical outcome and research on the underlying mechanisms are scarce (6). Identification of effective predictors could help to judge the prognosis and optimal intervention measures for COVID-19 patients at an early stage.

Therefore, 95 hospitalized COVID-19 pneumonia patients with definitive clinical outcome (cured or died) were retrospectively selected between February 5, 2020, and March 11, 2020. The clinical characteristics of the 95 hospitalized patients are described. Moreover, factors predicting the prognosis of patients with COVID-19 pneumonia were investigated in this study.

## Materials and Methods

### Study Design and Participants

This retrospective case-control study enrolled a total of 95 cases admitted to Tongji hospital between February 5, 2020, and March 8, 2020, with confirmed COVID-19 pneumonia. In brief, the patients were all hospitalized probable subjects in four wards of Tongji hospital with (1) positive throat swab nucleic acid test by real-time RT-PCR methods or positive for SARS-CoV-2-specific antibodies, (2) chest radiographic evidence of pneumonia, (3) rehabilitation discharge or died in hospital between February 20, 2020, and March 11, 2020. Patients who were discharged from hospital in 24 h or died within 24 h after hospitalization were excluded from this study. This study was reviewed and approved by the ethics committee of Tongji Hospital, Huazhong University of Science and Technology (IRB ID:TJ- IRB20200343).

### Data Collection

Clinical, laboratory, and radiological results were collected from electronic medical records. Data were obtained with standardized forms for all subjects involved. Two researchers independently collected and reviewed the data.

### Outcomes

Two outcomes were evaluated: “cured” or “died.” The criteria for rehabilitation discharge or being cured were (1) no fever for at least 3 days; (2) substantial improvement in chest CT scan or X-ray images; (3) negative nucleic acid test two consecutive times with at least a 24-h interval between them.

### Statistical Analysis

Descriptive analyses of the continuous variables were expressed as mean or median with interquartile range (IQR). Categorical variables were described as frequency rates and percentages. Differences in continuous variables were analyzed using *t*-tests when normally distributed or otherwise with the Mann-Whitney test. Categorical variables were compared using the χ^2^ test. The ROC curve was used to analyze the predictive factors, and the area under the ROC curve (AUC) was calculated. Correlation analysis was evaluated by the Pearson test. The appropriate sample size for inferences was determined based on the Wilcoxon statistics, where the statistical power was 0.8 (1–β) and α = 0.05. Forward stepwise, binary logistic regression was performed on the covariates. All tests were two-sided, and *P* < 0.05 was considered statistically significant. The statistical software SPSS 23.0 was used in this study.

## Results

### The Characterization of Patients

A total of 95 COVID-19 patients (58 men and 37 women) were included in the study (Table 1). The median age was 65 years old. As of March 11, 56 patients (58.9%) had been discharged, and 39 (41.1%) had died. The all-cause mortality rate in these COVID-19 patients was 22.4%. Of the 95 patients, 68 (71.6%) had one or more pre-existing diseases. Hypertension (40 [42.1%]), diabetes (22 [23.2.1%]), cardiovascular disease (10 [10.5%]), and malignancies (6 [6.3%]) were the most common. The chief complaints were fever (71 [74.7%]), cough (13 [13.7%]), dyspnea (6 [6.3%]), and other uncommon symptoms. Of the 95 patients, bilateral involvement was detected in chest CT or X-ray images of 92 (97%) patients. The mean hospitalization duration was 21 (14.0–25.0) days. People who died were significantly older (70 years [IQR 60–77] vs. 62 years [IQR 50.5–72]; *p* = 0.008), more likely to have malignancies (5 [13%] vs. 1 [2%]; *p* = 0.030), while other variables (i.e., gender, hypertension, diabetes, cardiovascular disease, COPD, hospital stay) were similar between the two groups.

**Table 1:** Baseline characteristics of patients included in the study.

	**Total** **(*n* = 95)**	**Cured** **(*n* = 56)**	**Died** **(*n* = 39)**	***P*-value**
Age, years	65 (56–76)	62 (50.5–72)	70 (60–77)	0.008
Sex				0.073
Male	58 (61%)	30 (54%)	28 (72%)	
Female	37 (39%)	26 (46%)	11 (28%)	
Any comorbidities	68 (72%)	37 (66%)	31 (79%)	0.154
Hypertension	40 (42%)	23 (41%)	17 (44%)	0.807
Diabetes	22 (23%)	12 (21%)	10 (26%)	0.632
Cardiovascular	10 (12%)	5 (9%)	5 (13%)	0.543
disease				
COPD	6 (6%)	3 (5%)	3 (8%)	0.645
Malignancy	6 (6%)	1 (2%)	5 (13%)	0.030
Chronic renal	7 (7%)	6 (11%)	1 (3%)	0.135
disease				
Others	15 (16%)	7 (13%)	8 (21%)	0.292
Chief complaint				
Fever	71 (75%)	42 (75%)	29 (74%)	0.944
Cough	11 (16%)	7 (13%)	6 (15%)	0.687
Dyspnea	6 (6%)	4 (7%)	2 (5%)	0.691
Others	5 (5%)	3 (5%)	2 (5%)	0.961
Bilateral involvement				0.142
Yes	92 (97%)	53 (95%)	39 (100%)	
No	3 (3%)	3 (5%)	0 (0%)	
Hospital stay, days	21.0 (14.0–25.0)	21.1 (15.0–27.0)	18.4 (13.0–22.0)	0.057

### Laboratory Parameters in the Cured and Died Groups

The blood count of patients on admission showed a decrease in lymphocytes, especially in the died group (Table 2). Consistent with this, most lymphocyte subsets (T cells, Th cells, Ts cells, and NK cells) detected by flow cytometry were significantly higher in survivors than in non-survivors. Meanwhile, leucocyte count and neutrophil count were higher in deceased patients (leucocyte 9.9 × 10^9^ per L [7.3–12.6]; neutrophil 8.6 × 10^9^ per L [6.1–11.4]) than in cured patients (leucocyte 8.6 × 10^9^ per L [6.1–11.4], *p* < 0.001; neutrophil 4.6 × 10^9^ per L [3.1–5.2], *p* = 0.001). Prothrombin time and D-dimer levels were higher in deceased patients (PT 16.1 s [14.3–15.8]; D-dimer 5.8 mg/L [1.4–6.8]) than in cured patients (PT 13.9 s [13.2–14.5], *p* < 0.001; D-dimer 2.8 mg/L [0.4–2.0], *p* = 0·001). Blood urea nitrogen levels, C-reactive protein (CRP) levels and Procalcitonin levels were higher in deceased patients (BUN 9.9 mmol/L [5.9–10.5]; CRP 123.2 mg/L [38.3–213.2]; Procalcitonin 1.3 ng/mL [0.1–0.6]) than in cured patients (BUN 5.9 mmol/L [3.7–6.4]), *p* < 0.001; CRP 43.5 mg/L [2.4–63.9], *p* = 0.006; Procalcitonin 0.2 ng/mL [0–0.1], *p* < 0.001). Serum albumin levels were lower in deceased patients (albumin 30.5 g/L [27.6–33.5]) than in cured patients (albumin 35.2 g/L [32.0–37.8], *p* = 0.022), while serum total bilirubin and lactate dehydrogenase levels were higher in deceased patients (T-Bil 13.3 μmol/L [9.8–17.6]; LDH 503.0 U/L [304.0–659.0]) than in cured patients (T-Bil 10.0 μmol/L [6.3–13.5], *p* = 0.010; LDH 305.4 U/L [212.5–348.3], *p* < 0.001), indicating hepatic dysfunction in more patients in the died group. The levels of most cytokines (IL2R, IL6, IL8, IL10, and TNF-α) were significantly higher in non-survivors than in survivors. No significant differences in serum creatine and BNP levels existed between deceased patients and cured patients (*p* > 0.05). As determined based on the Wilcoxon statistics (7), the sample size needed for evaluating CRP, Procalcitonin, LDH, IL6, neutrophil percentage, lymphocyte percentage, and T cells were 31 patients (18 cases cured, 13 cases died), 244 patients (144 cases cured, 100 cases died), 41 patients (24 cases cured, 17 cases died), 56 patients (33 cases cured, 23 cases died), 29 patients (17 cases cured, 12 cases died), 27 patients (16 cases cured, 11 cases died), and 103 patients (61 cases cured, 42 cases died), respectively.

**Table 2:** Laboratory findings of patients with COVID-19.

	**Total** (*n* = 95^*^)	**Cured** (*n* = 56^*^)	**Died** (*n* = 39^*^)	***P*-value**
Hemoglobin, g per L	126.0 (116.0–138.0)	122.3 (115.3–128.8)	131.5 (117.0–149.0)	0.009
Alanine aminotransferase, U/L	38.9 (15.0–44.0)	32.4 (14.0–43.3)	48.2 (18.0–46.0)	0.142
Aspartate aminotransferase, U/L	42.5 (21.0–45.0)	33.8 (19.3–41.3)	54.9 (24.0–56.0)	0.022
Albumin, g/L	33.3 (29.7–33.9)	35.2 (32.0–37.8)	30.5 (27.6–33.5)	<0.001
Total bilirubin, umol/L	11.4 (7.0–14.2)	10.0 (6.3–13.5)	13.3 (9.8–17.6)	0.010
Lactate dehydrogenase, U/L	386.6 (227.0–479.0)	305.4 (212.5–348.3)	503.0 (304.0–659.0)	<0.001
Blood urea nitrogen, mmol/L	7.6 (4.1–8.6)	5.9 (3.7–6.4)	9.9 (5.9–10.5)	0.002
Creatinine, umol/L	119.4 (59.0–98.0)	115.7 (56.0–92.8)	124.7 (65.0–120.0)	0.796
Prothrombin time, second	14.8 (13.5–15.1)	13.9 (13.2–14.5)	16.1 (14.3–15.8)	<0.001
D–dimer, ug/mL	4.0 (0.6–4.4)	2.8 (0.4–2.0)	5.8 (1.3–6.8)	0.001
Platelets, × 10^9^ per L	219.1 (147.0–291.0)	232.1 (171.8–304.5)	200.4 (121.0–255.0)	0.137
Procalcitonin, ng/mL	0.7 (0.0–0.4)	0.2 (0–0.1)	1.3 (0.1–0.6)	<0.001
C–reactive protein, mg/L	69.4 (3.4–105.4)	43.5 (2.4–63.9)	123.2 (38.3–213.2)	0.006
BNP, pg/mL	1739.1 (95.5–1274.8)	1653.4 (67.0–457.0)	1848.0 (413.5–1472.0)	0.859
IL2R, U/mL	998.4 (499.5–1390.1)	851.5 (413.0–1250.0)	1188.9 (861.0–1522.0)	0.010
IL6, pg/mL	79.3 (4.4–65.7)	31.9 (2.6–38.5)	139.1 (21.3–146.8)	<0.001
IL8, pg/mL	28.6 (8.6–33.9)	18.5 (7.1–22.7)	41.7 (16.5–48.9)	<0.001
IL10, pg/mL	5.7 (0–8.4)	3.2 (0–5.4)	8.8 (0–10.1)	0.036
TNF–α, pg/mL	10.9 (6.9–13.7)	9.3 (5.7–12.2)	13.1 (8.3–15.6)	0.009
Leucocytes, × 10^9^ per L	7.9 (5.1–9.0)	6.5 (4.6–7.5)	9.9 (7.3–12.6)	<0.001
Neutrophils, × 10^9^ per L	6.3 (3.5–7.6)	4.6 (3.1–5.2)	8.6 (6.1–11.4)	<0.001
Neutrophil percentage, %	76.1 (66.8–87.1)	69.2 (61.2–75.0)	85.9 (80.8–91.6)	<0.001
Lymphocytes, × 10^9^ per L	1.0 (0.6–1.4)	1.2 (0.7–1.6)	0.7 (0.3–0.7)	<0.001
Lymphocyte percentage, %	15.3 (6.9–22.6)	20.5 (14.9–27.0)	7.9 (4.6–9.9)	<0.001
Monocytes, × 10^9^ per L	0.5 (0.3–0.6)	0.5 (0.4–0.6)	0.5 (0.3–0.7)	0.850
Eosinophils, × 10^9^ per L	0.1 (0–0.1)	0.1 (0–0.1)	0 (0–0)	0.056
Basophils, × 10^9^ per L	0 (0–0)	0 (0–0)	0 (0–0)	0.184
T cells (CD3+CD19–) /ul	556.9 (206.3–880.3)	1073.3 (842.0–1499.3)	322.2 (125.0–471.3)	<0.001
T cells (CD3+CD19–) %	14.3 (56.1–78.8)	74.9 (68.3–80.4)	64.0 (54.4–75.0)	0.042
B cells (CD3–CD19+) /ul	115.4 (41.5–150.3)	114.1 (49.8–167.5)	116.0 (36.8–146.8)	0.958
B cells (CD3–CD19+) %	19.2 (9.4–29.6)	8.9 (4.1–11.4)	23.9 (10.5–33.4)	0.005
Th cells (CD3+CD4+) /ul	369.8 (107.3–587.0)	707.5 (505.0–924.5)	216.4 (85.3–239.3)	<0.001
Th cells (CD3+CD4+) %	44.3 (31.7–57.3)	49.4 (43.1–58.7)	42.0 (30.4–57.5)	0.189
Ts cells (CD3+CD8+) /ul	168.5 (53.3–265.0)	327.6 (256.3–365.8)	96.2 (32.5–131.5)	<0.001
Ts cells (CD3+CD8+) %	21.1 (12.2–23.1)	22.7 (17.2–27.9)	20.4 (11.3–21.3)	0.661
NK cells (CD3–/CD16+ CD56+) /ul	104.1 (26.5–121.0)	223.3 (99.8–293.5)	49.9 (15.0–69.8)	<0.001
NK cells (CD3–/CD16+ CD56+) %	12.3 (6.3–16.0)	14.8 (6.7–17.0)	11.1 (4.9–16.2)	0.326
T cells+B cells+NK cells /ul	776.5 (98.6–1146.5)	1410.7 (1118.3–1902.5)	488.2 (262.8–713.3)	<0.001
T cells+B cells+NK cells %	98.9 (98.6–99.5)	98.7 (98.1–99.5)	99.0 (98.7–99.5)	0.329
Th/Ts	2.9 (1.6–3.9)	2.4 (1.7–2.9)	3.1 (1.5–4.5)	0.304

* *C-reactive protein levels were tested in 46 patients (31 cases cured, 15 cases died), cytokine levels were tested in 85 patients (48 cases cured, 37 cases died), while lymphocyte subgroups were tested in 32 patients (10 cured, 22 died)*.

*INR, International standardized ratio; Th cells, helper T cells; Ts cells, suppressor T cells; NK cells, natural killer cells. Continuous variables are described as mean (IQR)*.

### Prognostic Factors of Patients With COVID-19 Pneumonia

**Figure 1:**
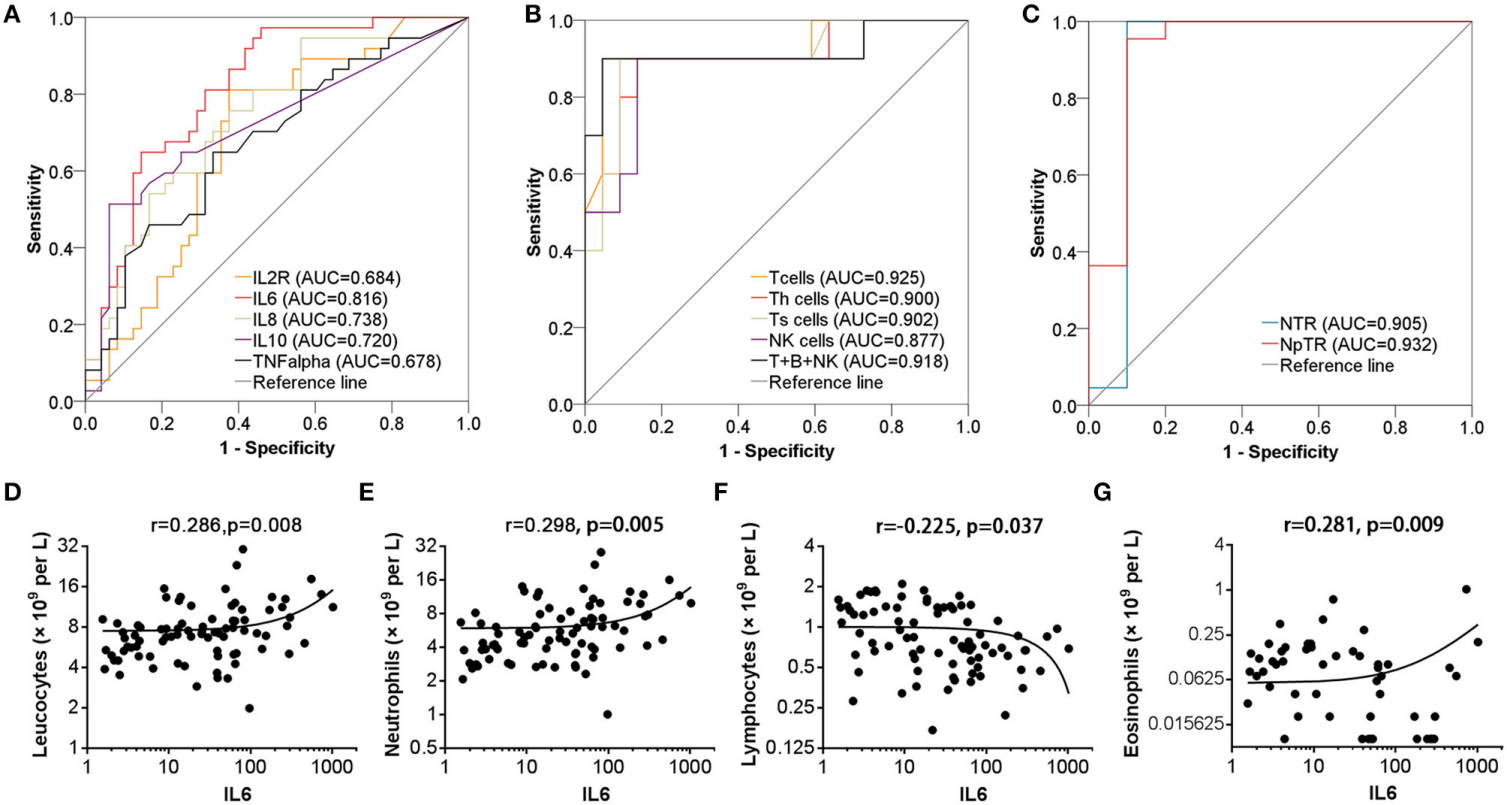
Efficiency of cytokine and leucocyte subsets in predicting the clinical outcome of patients with COVID-19 pneumonia. **(A–C)** Efficiency of serum cytokine and leucocyte subsets in predicting the mortality of patients. **(D–G)** Serum IL6 levels were positively correlated with lymphocyte count, neutrophil count, and eosinophil count and negatively correlated with lymphocyte count.

ROC curve analysis was performed to evaluate the prognostic factors for COVID-19. The neutrophil-to-lymphocyte ratio (NLR) is a well-known marker of systemic inflammation (8). Therefore, we evaluated the efficiency of NLR and other potential predictors (neutrophil to T lymphocyte count ratio, NTR; neutrophil percentage to T lymphocyte ratio, NpTR) in predicting mortality. As shown in Table 3 and Figures 1A–C, factors associated with peripheral blood cell count (neutrophils, neutrophil percentage, lymphocyte percentage, T cells, B cells, Th cells, Ts cells, NK cells, T cells+B cells+NK cells, NLR, NTR, and NpTR) and inflammation-associated factors (Procalcitonin, IL6) showed good prognostic values, among which NpTR was the most predominant predictive factor for the clinical outcome (0.932; 95% CI: 0.810–1.000, *p* < 0.001; Figure 1C). Furthermore, binary logistic regression models showed that NpTR was the independent prognostic factor for death (OR =59993.937, 95% CI: 4.130–871565732.1; *p* = 0.024; Table 4). The Nagelkerke R value was 0.811. The condition indexes for age, LDH, and NpTR were 2.6, 5.4, and 18.6, respectively. To minimize potential confounding effects of age, a matched case-control study was performed (each deceased patient was matched with one cured patient with an age difference of 4 years or less). Both the matched and unmatched analyses yielded similar results (Supplementary Table 1).

**Table 3:** Prognostic value of the clinical parameters.

	**Number**	**ACU**	**95% Confidence interval**	***P*-value**
Age	95	0.658	0.549–0.768	0.009
Malignancy	95	0.555	0.435–0.675	0.362
Hemoglobin, g per L	95	0.657	0.537–0.777	0.009
Aspartate aminotransferase	95	0.638	0.525–0.751	0.022
Albumin	95	0.739	0.637–0.840	<0.001
Total bilirubin	95	0.695	0.591–0.800	0.001
Lactate dehydrogenase	95	0.784	0.689–0.879	<0.001
Blood urea nitrogen	95	0.782	0.687–0.877	<0.001
Prothrombin time	95	0.772	0.671–0.873	<0.001
D–dimer	95	0.708	0.598–0.819	0.001
Procalcitonin	95	0.822	0.734–0.910	<0.001
C–reactive protein	46	0.759	0.610–0.907	0.007
IL2R	85	0.684	0.570–0.798	0.004
IL6	85	0.811	0.721–0.901	<0.001
IL8	85	0.738	0.631–0.845	<0.001
IL10	85	0.720	0.606–0.835	0.001
TNFa	85	0.678	0.562–0.793	0.005
Leucocytes	95	0.798	0.702–0.895	<0.001
Neutrophils	95	0.851	0.766–0.937	<0.001
Neutrophil percentage	95	0.900	0.837–0.964	<0.001
Lymphocytes	95	0.777	0.683–0.870	<0.001
Lymphocyte percentage	95	0.897	0.833–0.962	<0.001
Monocyte	95	0.521	0.395–0.647	0.728
Eosinophils	95	0.755	0.655–0.855	<0.001
Basophils	95	0.554	0.437–0.670	0.376
T cells (CD3+CD19–)	32	0.925	0.808–1.000	<0.001
T cells (CD3+CD19–)%	32	0.732	0.543–0.920	0.038
B cells (CD3–CD19+)%	32	0.805	0.653–0.956	<0.001
Th cells (CD3+CD4+)	32	0.900	0.771–1.000	<0.001
Ts cells (CD3+CD8+)	32	0.902	0.776–1.000	<0.001
NK cells (CD3–/CD16+CD56+)	32	0.877	0.731–1.000	0.001
T cells+B cells+NK cells	32	0.918	0.780–1.000	<0.001
NLR	32	0.900	0.837–0.964	<0.001
NTR	32	0.905	0.727–1.000	<0.001
NpTR	32	0.932	0.810–1.000	<0.001

*NLR, neutrophil-to-lymphocyte ratio; NTR, neutrophil to T lymphocyte count ratio; NpTR, neutrophil percentage to T lymphocyte ratio; Th cells, helper T cells; Ts cells, suppressor T cells; NK cells, natural killer cells*.

**Table 4:** Logistic regression of independent prognostic factors for mortality.

**Factors**	**β**	**S$\bar{x}$**	***P*-value**	**OR for Mortality (95% CI)**
Age	0.134	0.108	0.214	1.143 (0.925–1.412)
PT	2.334	1.258	0.064	10.323 (0.877–121.5630.690)
NpTR	11.002	4.890	0.024	59993.937 (4.130–871565732.1)

*NpTR, neutrophil percentage to T lymphocyte ratio; PT, Prothrombin time*.

### Correlations Between Leucocyte Subsets and Inflammation-Related Factors

We subsequently explored the correlations between leucocyte subsets and inflammation-related factors (IL2R, IL6, IL8, TNF-α, CRP, and Procalcitonin) associated with clinical outcome. Interestingly, serum IL6 levels were positively correlated with leucocyte count, neutrophil count, and eosinophil count (*r* = 0.286, *p* = 0.008; *r* = 0.298, *p* = 0.005; *r* = 0.281, *p* = 0.009, resp.) and negatively correlated with lymphocyte count (*r* = −0.226, *p* = 0.037) but not monocyte count, as shown in Table 5 and Figures 1D–G. Meanwhile, leucocytes, neutrophils, and eosinophils were positively correlated with most inflammatory factors (IL2R, IL6, IL8, TNF-α, CRP, and Procalcitonin), as shown in Table 5. No significant correlations were found between lymphocyte subsets and inflammatory factors (IL2R, IL6, IL8, TNF-α, and Procalcitonin) (*p* > 0.05).

**Table 5:** Correlation analysis was evaluated between leucocyte subsets and inflammatory factors.

	**IL2R**	**IL6**	**IL8**	**IL10**	**TNF-α**	**CRP**	**PCT**
Leucocytes	0.232^*^	0.286^**^	0.382^**^	0.474^**^	0.126	0.440^**^	0.196
Neutrophils	0.247^*^	0.298^**^	0.403^**^	0.474^**^	0.123	0.500^**^	0.177
Lymphocytes	−0.218^*^	−0.225^*^	−0.226^*^	−0.185	−0.147	−0.422^**^	−0.025
Monocyte	0.084	0.120	−0.007	0.111	0.107	0.159	−0.004
Eosinophils	0.203^*^	0.281^**^	0.234^*^	0.463^**^	0.390^**^	−0.348^*^	0.667^**^
T cells	−0.165	0.035	−0.021	0.010	−0.188	−0.316	0.138
B cells	0.091	0.093	0.068	0.053	−0.048	0.486^*^	0.028
Th cells	−0.085	0.123	0.025	0.075	−0.173	−0.236	0.202
Ts cells	−0.279	−0.136	−0.103	−0.127	−0.176	−0.374	−0.020
NK cells	−0.321	−0.231	−0.208	−0.195	−0.119	−0.531^*^	−0.088
T +B +NK cells	−0.199	−0.014	−0.057	−0.031	−0.190	−0.325	0.094

*Data expressed as correlation coefficient*.

* P < 0.05;

** *P < 0.01. NK cells, natural killer cells; Th cells, helper T cells; Ts cells, suppressor T cells; CRP, C-reactive protein; PCT, Procalcitonin*.

## Discussion

We retrospectively analyzed the clinical characteristics and laboratory parameters in 95 COVID-19 patients with definitive clinical outcome and found that leucocyte subsets and inflammatory factors showed good prognostic values and that non-self-limiting inflammatory response may be involved in the development of fatal pneumonia induced by SARS-CoV-2 infection.

As previously reported, older age, a coagulation disorder, bacterial infection, malfunctions in the liver, heart, or kidney, and changes in blood cell count are associated with the prognosis of COVID-19 patients (4, 6). Similarly, we found that older age, lower albumin, and higher serum LDH levels, BUN levels, and PT indicated poor outcome. Patients with increased levels of organ damage-associated biomarkers were more likely to develop complications such as fetal acute lung injury (ALI) and multiple organ dysfunction syndrome. Moreover, the leucocyte count or neutrophil count was higher in the died group and predicted the mortality in COVID-19 patients, verified by ROC curves, while lymphocyte subset (lymphocytes, T cells, Th cells, Ts cells, NK cells, T cells+B cells+NK cells) counts were positively associated with cure rate, among which the AUC for T cells was the highest (AUC: 0.925, *P* < 0.001). NLR is a well-known marker of systemic inflammation (8), so we evaluated NLR and other potential predictors (NTR, NpTR) originating from the existing parameters. ROC curves showed that NLR, NTR, and NpTR could effectively predict the mortality in patients with COVID-19 pneumonia (AUC: 0.900, *P* < 0.001; AUC: 0.905, *P* < 0.001; AUC: 0.932, *P* < 0.001, resp.).

Despite a series of studies, specific factors causing the high mortality of COVID-19 are incompletely understood. Respiratory failure is the main cause of mortality in COVID-19 patients (4, 9). Severe pneumonia caused by pathogenic human coronavirus is often associated with high viral load, massive inflammatory cell infiltration, and elevated cytokine responses resulting in ALI (10, 11). Consistent with the above findings, histological examination of lungs from patients dead from COVID-19 revealed extensive leucocyte infiltration, overactivated T lymphocytes being the predominant cell type (9). Hyper-inflammatory cytokines can amplify inflammatory responses by promoting unrestrained virus replication (12). Furthermore, coronavirus-infected patients may die of ALI despite successful viral elimination (13). Consistent with previous studies (4, 14), we found that higher levels of inflammation-associated factors (IL2R, IL6, IL8, TNF-α, CRP, and Procalcitonin) indicated poor outcome, indicating that powerful positive feedback between virus infection and hyperinflammation might be critical in lung destruction and disease morbidity. IL6 was a powerful predictor of mortality and closely correlated with leucocyte, neutrophil, lymphocyte, and eosinophil counts. Thus, IL6 receptor blockade (tocilizumab), which has been approved for a clinical COVID-19 treatment trial in China (ChiCTR2000029765), is a new and promising treatment for COVID-19. IL-10 was highly expressed in non-survivors, indicating the failure to limit and ultimately terminate hyperinflammation in severe patients (15). Meanwhile, lymphocytes play an important role in SARS-CoV-2 elimination, as indicated by the production of larger amounts of granzymes and perforin in a mild-moderate patient (16). There was no significant elevation of cytokines in this mild-moderate patient, further indicating the key role of cytokines in disease progression. Middle East respiratory syndrome coronavirus (MERS) can efficiently infect human T cells and induced apoptosis in human T lymphocytes (17), so it is speculated that both redistribution to the target organ and depletion contribute to lymphocyte decline, both of which indicate poor outcome. Studies have demonstrated that T-cell responses can inhibit the overactivation of innate immunity (18). As described in our study, leucocytes, neutrophils, and eosinophils were positively correlated with most inflammatory factors (IL2R, IL6, IL8, TNF-α, CRP, and Procalcitonin), indicating that neutrophils and eosinophils might contribute to cytokine release. Meanwhile, lymphocyte count significantly decreased in fatal cases and was negatively correlated with IL6 levels, indicating negative feedback between lymphocytes and IL6 during coronavirus infection. Dysfunction of lymphocytes in both virus clearance and inhibition of cytokine overactivation may contribute to excessive inflammatory response. Thus, non-self-limiting inflammatory response and lymphocyte dysfunction may be the key mechanisms in fatal pneumonia induced by SARS-CoV-2 infection. Probably because of the limited number of cases detected, no correlations were found between lymphocyte subsets and inflammatory factors.

This study does have some limitations. Firstly, owing to the retrospective nature of this study, results for viral load not available. Secondly, it is a single-center study with a limited number of cases. In addition, the majority of patients admitted to our hospital were critically ill, so population bias exists, though a confounding effect of age has been ruled out.

## Conclusion

In summary, our results demonstrate that leucocyte subsets and inflammation-related factors predict the clinical outcome of patients with COVID-19 pneumonia with high efficiency, among which T cells and NpTR are most predominant. Non-self-limiting inflammatory response and lymphocyte dysfunction may be the key mechanisms in fatal pneumonia induced by SARS-CoV-2 infection. This provides evidence for laboratory diagnostics and clinical interventions.

## Data Availability Statement

The raw data supporting the conclusions of this article will be made available by the authors, without undue reservation.

## Ethics Statement

The studies involving human participants were reviewed and approved by The Ethics Committee of Tongji Hospital of Huazhong University of Science and Technology. Written informed consent for participation was not required for this study in accordance with the national legislation and the institutional requirements.

## Author Contributions

SL and CY made substantial contributions to the study design. JG and CY were in charge of the draft manuscript. JL and SL took responsibility for data acquisition. JL made the main contributions to data analysis and interpretation. CY participated in the diagnosis and treatment of patients. CY made substantial revisions to the manuscript. All authors contributed to the article and approved the submitted version.

## Conflict of Interest

The authors declare that the research was conducted in the absence of any commercial or financial relationships that could be construed as a potential conflict of interest.
