# Supplementary material for: Leucocyte Subsets Effectively Predict the Clinical Outcome of Patients With COVID-19 Pneumonia: A Retrospective Case-Control Study
Source: Front Public Health. 2020 Jun 18;8:299. doi: 10.3389/fpubh.2020.00299 (PMC7314901; doi:10.3389/fpubh.2020.00299)
Supplement: Supplementary file 1 [file Table_1.doc]

|  | | **Numbers** | **ACU** | **95% Confident interval** | **P value** |
| --- | --- | --- | --- | --- | --- |
| Age | 78 | | 0.531 | 0.400-0.661 | 0.642 |
| Malignancy | 78 | | 0.551 | 0.423-0.680 | 0.436 |
| Hemoglobin, g per L | 78 | | 0.660 | 0.535-0.786 | 0.015 |
| Aspartate aminotransferase | 78 | | 0.621 | 0.496-0.747 | **0.065** |
| Albumin | 78 | | 0.716 | 0.601-0.831 | 0.001 |
| Total bilirubin | 78 | | 0.640 | 0.517-0.764 | 0.033 |
| Lactate dehydrogenase | 78 | | 0.760 | 0.651-0.869 | <0.001 |
| Blood urea nitrogen | 78 | | 0.763 | 0.656-0.870 | <0.001 |
| Prothrombin time | 78 | | 0.753 | 0.643-0.863 | <0.001 |
| D-dimer | 78 | | 0.720 | 0.604-0.836 | 0.001 |
| Procalcitonin | 78 | | 0.817 | 0.679-0.954 | 0.001 |
| C-reactive protein | 37 | | 0.748 | 0.588-0.909 | 0.012 |
| IL2R | 72 | | 0.683 | 0.557-0.809 | 0.008 |
| IL6 | 72 | | 0.778 | 0.670-0.887 | <0.001 |
| IL8 | 72 | | 0.723 | 0.605-0.840 | 0.001 |
| IL10 | 72 | | 0.702 | 0.579-0.826 | 0.003 |
| TNFa | 72 | | 0.698 | 0.577-0.819 | 0.004 |
| Leucocytes | 78 | | 0.826 | 0.728-0.925 | <0.001 |
| Neutrophils | 78 | | 0.855 | 0.765-0.945 | <0.001 |
| Neutrophil percentage | 78 | | 0.863 | 0.777-0.949 | <0.001 |
| Lymphocytes | 78 | | 0.716 | 0.602-0.831 | 0.001 |
| Lymphocyte percentage | 78 | | 0.753 | 0.638-0.868 | <0.001 |
| Monocyte | 78 | | 0.521 | 0.388-0.654 | 0.749 |
| Eosinophils | 78 | | 0.751 | 0.640-0.862 | <0.001 |
| Basophils | 78 | | 0.529 | 0.400-0.658 | 0.660 |
| T cells (CD3+CD19-) | 31 | | 0.917 | 0.788-1.000 | <0.001 |
| T cells (CD3+CD19-) % | 31 | | 0.707 | 0.507-0.907 | **0.074** |
| B cells (CD3-CD19+) % | 31 | | 0.808 | 0.654-0.962 | 0.008 |
| Th cells (CD3+CD4+) | 31 | | 0.889 | 0.748-1.000 | 0.001 |
| Ts cells (CD3+CD8+) | 31 | | 0.891 | 0.753-1.000 | 0.001 |
| NK cells (CD3-/CD16+CD56+) | 31 | | 0.874 | 0.716-1.000 | 0.001 |
| T cells+B cells+NK cells | 31 | | 0.909 | 0.757-1.000 | <0.001 |
| NLR | 31 | | 0.828 | 0.613-0.964 | 0.005 |
| NTR | 31 | | 0.894 | 0.698-1.000 | 0.001 |
| NpTR | 31 | | 0.924 | 0.790-1.000 | <0.001 |

**Supplementary Table 1. Prognostic value of the clinical parameters in the matched case-control study.**

Notes: NLR, neutrophil-to-lymphocyte ratio; NTR, neutrophil to T lymphocyte count ratio; NpTR, neutrophil percentage to T lymphocyte ratio; Th cells, helper T cells; Ts cells, suppressor T cells; NK cells, natural killer cells.
